# MOF the beaten track: unusual structures and uncommon applications of metal–organic frameworks

**DOI:** 10.1186/s13065-017-0330-0

**Published:** 2017-10-11

**Authors:** Alexander J. Tansell, Corey L. Jones, Timothy L. Easun

**Affiliations:** School of Chemistry, Cardiff University, Main Building, Park Place, Cardiff, CF10 3AT UK

**Keywords:** Metal–organic framework, Host–guest chemistry, Post-synthetic modification, Functional materials

## Abstract

Over the past few decades, metal–organic frameworks (MOFs) have proved themselves as strong contenders in the world of porous materials, standing alongside established classes of compounds such as zeolites and activated carbons. Following extensive investigation into the porosity of these materials and their gas uptake properties, the MOF community are now branching away from these heavily researched areas, and venturing into unexplored avenues. Ranging from novel synthetic routes to post-synthetic functionalisation of frameworks, host–guest properties to sensing abilities, this review takes a sidestep away from increasingly ‘traditional’ approaches in the field, and details some of the more curious qualities of this relatively young family of materials.

## Introduction

With over 2000 new papers in the field entering the literature every year^1^ metal–organic frameworks (MOFs) are an increasingly well-studied and, in some areas, well understood subset of porous materials. Within the MOF literature, the most commonly described potential applications of these materials are based on their impressive gas storage and sorption properties. Exploration into their capabilities is rapidly expanding, with an increasing number of reviews in areas which describe different aspects of MOFs such as: flexibility [1, 2], guest adsorption [3], stimuli-response [4], hybridity [5], photoresponse [6, 7], catalysis [8], sensing [9], polymerisation vessels [10], mechanochromic luminescent properties [11], applications of nanoscalability [12], use in batteries and supercapacitors [13], uses as nanomedicine platforms [14], defects and defect engineering [15, 16], computation prediction [17], surface chemistry [18] and manipulation into gels [19]. In this review, we have selected metal–organic frameworks and MOF applications that are outside their traditional and well-reviewed areas, but which further demonstrate the enormously broad potential of this class of materials. Some of the chosen articles are well-known in their respective areas, but we have endeavoured to find those works which have perhaps not yet received the attention they deserve.

## Synthesis of MOF materials

**Fig. 1:**
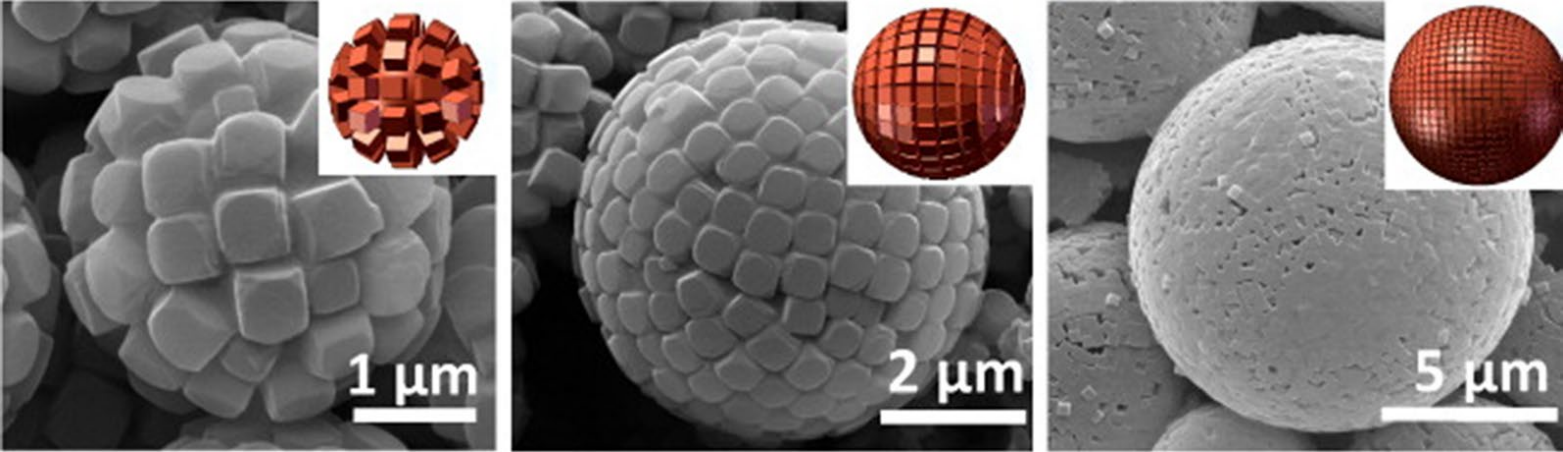
SEM images of colloidosomes formed from cubic Fe-soc-MOF building blocks (Reprinted with permission from Ref [29]. Copyright 2013 American Chemical Society)

In recent years, a more rigorous understanding of design criteria and structure–function relationships has begun to emerge. The principles of directed assembly are becoming increasingly important—both in linker design and in synthetic methodology, and in particle morphology. Until recently, the notable features of a metal–organic framework have often been attributed to its function, with less consideration given to the methods of preparation. However, there are a growing number of interesting reports that intrinsically link function with variations in synthetic approach, which can result in, for example, markedly different particle sizes. Given that MOFs are traditionally synthesised as crystalline materials, the approach of Angulo-Ibáñez et al. to the synthesis of Co(II)- and Ni(II)-based metal–organic gels, dubbed “metallogels”, and their subsequent aerogel and xerogel analogues, is a significant departure from that tradition [20]. One of the main advantages of this type of synthesis is the pore size of these aerogel products exceeds that of MOFs, allowing them to interact with larger molecules leading to many new applications. These synthesis methods also inspired work by Ruiz-Pérez and co-workers to investigate the polymorphic control of Eu(III) frameworks through gel and hydrothermal methods [21]. Microwave-assisted synthesis of frameworks is also of increasing prevalence. Taddei et al. have described the UiO-66 MOF prepared by microwave-assisted synthesis, where improved consistency was displayed in crystal size, defects and morphology compared to those prepared by conventional heating [22]. Bag et al. have explored the advantages of microwave-assisted large scale synthesis, where the syntheses of a series of nanoscale luminescent lanthanide frameworks were reported [23]. The rapid synthesis of MIL-53(Al) was reported by Laybourn and co-workers, who detailed the synthesis of 62 mg of framework in 4.3 s [24]. Aside from directly comparing successes of microwave-assisted synthesis compared to conventional synthesis, Schröder, Kingman and co-workers explored the effect that the dielectric constant of MOF reagents had on their solubility in microwave-assisted synthesis [25]. Lin et al. have adopted an alternative microwave synthetic approach whereby, instead of conventional solvents, they have used ionic liquids (ILs) to produce two anionic MOFs [26]. ILs are gaining popularity as potentially green solvents; 1-ethyl-3-methylimidazolium bromide (EMIm-Br) was chosen here to act as a template during the reaction. The products, (EMIm)_2_[Ni_3_(TMA)_2_(OAc)_2_] and (EMIm)_2_[Co_3_(TMA)_2_(OAc)_2_], were formed in a microwave-assisted reaction at 200 °C for 50 min under ionothermal conditions, and they were found to be isostructural. Ionothermal synthesis was well reviewed by Parnham et al. in 2007 [27], and utilised more recently in 2016 by Xu, Jiao and co-workers in the preparation of a series of Co-based frameworks [28]. Eight different ionic liquids were investigated as the reaction solvent based on 1-methyl-3-alkylimidazolium halide, and it was found that all the frameworks synthesised exhibited the same topologies. Eddaoudi, Zeng and co-workers employed a variety of synthetic approaches in the construction of hollow superstructures, or “colloidosomes”, from cubic fundamental MOF building blocks around an emulsion droplet [29]. They termed these building blocks Fe-soc-MOF cubes and the images shown in Fig. 1 illustrate how the size of the superstructures constructed was easily controlled by the size of the emulsion droplet. Colloidosomes are often constructed from spherical silica or polystyrene beads, so the sorption properties of the MOF-based colloidosome can be further controlled by the intrinsic porous nature of the Fe-soc-MOF building blocks. The control of particle and macroscale structure of MOFs is discussed further below.

## Nanoscale structural control

### Interpenetration

**Fig. 2:**
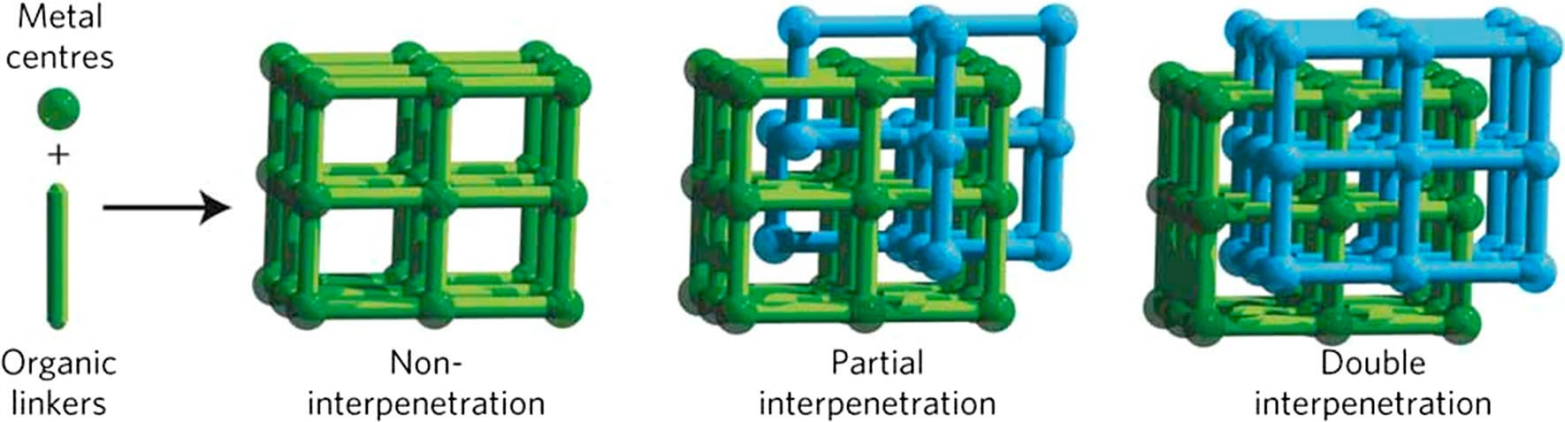
NOTT-202 is composed of one dominant network (green) and one secondary partially formed network (blue) resulting in a unique partially interpenetrated framework (Reprinted by permission from Macmillan Publishers Ltd: Nature Materials, Ref [38], copyright 2012)

Interpenetration is a common feature in MOFs which can affect the size and shape of the pores within a framework structure. Multiple lattices can become entwined leading to varying degrees of interpenetration, with examples ranging from two- to ten-fold. Typically, the more interpenetrated structures show greater stability and rigidity, but lower overall porosity [30]. However, in 2014, Nandi and Vaidhyanathan described a threefold interpenetrated MOF, Zn_2_(OOC–C_5_H_4_N)_4_(DMF) (DMF = dimethylformamide) that displays a higher porosity (~ 18.5%) than a related non-interpenetrated zinc isonicotinate MOF. The synthesis conditions were modified to produce the lower symmetry interpenetrated and more open framework. Gas sorption studies of the post-combustion porous carbons formed from these MOFs were performed at 77 K for nitrogen adsorption and 273 K for carbon dioxide adsorption. The results showed a N_2_ uptake of 20 mmol/g and a CO_2_ uptake of 3.5 mmol/g, compared with no N_2_ uptake and 1.5 mmol/g CO_2_ uptake of the related zinc isonicotinate MOF [31]. Ren et al. have reported a structural transformation via solvent-mediated anion exchange in three luminescent MOFs, [Cd(BCbpy)(BDC)]·3H_2_O, [Cd_2_(BCbpy)_2_(BDC)Cl_2_][Cd(BCbpy)_2_(BDC)]·18H_2_O and [Cd(BCbpy)Cl_2_]·3H_2_O (BCbpy = 1-(4-carboxybenzyl)-4,4-bipyridinium, BDC = 1,4-benzenedicarboxylic acid) [32]. These interpenetrated Cd(II) frameworks consist of BDC^2−^ ligands which can be exchanged in situ with anions, such as Cl^−^, leading to simplification of the complicated topology in which non-interpenetrated networks are formed, which can be observed by fluorescence switching. Interpenetration can sometimes pose a problem when porosity is needed as a function so several attempts to reduce, control and avoid this issue have been reported. In 2015, the control of the degree of interpenetration in a Mn framework, [Mn(SCN)_2_L_2_]_n_ where L are bis(4-pyridyl) substituted hydrazine subunits, was explored [33]. When the ligand cannot form hydrogen bonds with solvents, an interpenetrated structure is formed with pores of each 2D network occupied by atoms of two adjacent networks. Addition of an amide group in the linker that can form H-bonds with solvents prevents an interpenetrated network from forming. Wang et al. have synthesised five new zinc and cadmium frameworks with a “V-shaped” BPPA (BPPA = bis(4-(pyridine-4-yl)phenyl)amine) linker, which crystallises with a variety of co-linkers that control the degree of interpenetration. Notably, TFBDC (TFBDC = 2,3,5,6-tetrafluoroterephthalic acid) was used as a co-linker in the formation of {[Zn_3_(BPPA)_3_(TFBDC)_3_]·H_2_O}_n_ and {[Cd_2_(BPPA)_2_(TFBDC)_2_]}_n_ in which they found that interpenetration was successfully avoided to afford a rare 3D *6T8* topology [34]. Another successful approach to avoiding interpenetration was that reported by Chang et al. where they synthesised UTSA-68, [Cu_2_BTPC(H_2_O)_2_·(DMF)_2_·(H_2_O)] where BTPC = biphenyl-3,3′,5,5′-tetra-(phenyl-4-carboxylic) acid, by varying reaction conditions to give a 3D porous structure, leading to increased C_2_H_2_/CO_2_ gas separation when compared to the doubly interpenetrated MOF (ZJU-30a) [35]. The use of temperature to control the degree of interpenetration was explored by Barbour and co-workers, whereby a cobalt framework, [Co_2_(ndc)_2_(4,4′-bpy)] where ndc = 2,6-napthalene dicarboxylate and 4,4′-bpy = 4,4′-bipyridyl, undergoes a conversion from doubly-interpenetrated to triply-interpenetrated when activated at 120 °C [36]. Interpenetration can lead to increased framework stability at the cost of porosity, and the less common partial interpenetration is an interesting conceptual compromise in which one sub-lattice is fully occupied and the other exhibits partial occupancy. There are relatively few well-characterised examples in the literature, and control of the phenomenon is very limited. In 2016, Ferguson et al. reported the control of partial interpenetration in MUF-9 during its synthesis by varying reaction time and solvent composition [37]. Figure 2 shows a partially interpenetrated framework reported by Schröder et al. in 2012. NOTT-202, (Me_2_NH_2_)_1.75_[In(BTPC)]_1.75_(DMF)_12_(H_2_O)_10_, exhibits a structure change on increasing pressure of CO_2_ that leads to marked adsorption/desorption hysteresis. This could be considered an example of a flexible framework, although flexibility was proposed to arise from the movement of the partial net within the complete net, rather than from any significant structure changes within the framework sub-lattices [38].

Flexible MOFs have become increasingly prevalent in the literature in recent years [2], leading to Zhou et al. in 2015 reporting a series of isostructural interpenetrated frameworks, [Ag_6_(μ_8_-X)(Rtz)_4_]OH·6H_2_O where X = Cl, Br and Rtz = atz^−^ or mtz^−^ (Hatz = 3-amino-1,2,4-triazole and Hmtz = 3-methyl-1,2,4-triazole) that can be transformed by interpenetration reconstitution, in which the MOF can alter their metal-linker connectivity forming a network that is unattainable via direct synthesis [39]. This process was found to occur in the presence of water molecules or hydroxide ions as they are able to attack the Ag ions. By adjusting the hydrophobicity or hydrophilicity of the linker substituent groups can suppress this process. These groups control the guest accessibility to the open metal sites, determining which bonds can be easily broken for rearrangement of the interpenetration. The unusual flexibility of these materials also leads to them exhibiting rare water sorption properties.

### Defects

Defects can be engineered (both deliberately and serendipitously) in MOFs to produce materials with improved function for adsorption, catalysis, etc. This was demonstrated recently using UiO-66 as an example by Thornton et al., whereby the relationship between CO_2_ adsorption and mechanical stability was studied computationally [40]. The authors concluded that there is a compromise in the framework stability when defects are used to improve the adsorption, much as compromises are inherent in interpenetrated or partially interpenetrated structures. However, the stability of the defects can be preserved by further engineering of the different types of defects and their distribution through a structure [41]. The effect that defect engineering has on the flexibility of a framework was investigated by Hobday et al., who substituted the 4,4′-biphenyl dicarboxylate (BPDC) linker present in UiO-67 with 4,4′-azobenzene dicarboxylate (abdc), to form UiO-abdc [42]. When loaded with methanol in a diamond anvil cell, no compression of either material was observed when pressurised. This resilience was attributed to disorder within the linker systems. Whilst exhibiting local disorder, abdc also appears to bow in and out of the horizontal plane, which increases the flexibility of the framework. The zero-compressibility of UiO-67 was attributed to the large elastic modulus of the framework, reducing structural change during compression.

### Polymorphism

An example of how polymorphism in MOFs can affect the uptake of gases has been described by Zhu et al. [Cu_3_(BTEB)_2_(H_2_O)_3_], where BTEB = 1,3,5-benzene-trisethynylbenzoic acid, was found to have two topologies (*pto* and *tbo*), both based around a Cu-paddlewheel [43]. During the synthesis of these frameworks, the addition of 4,4′-bipyridine as a topological modifier led to the formation of the *pto* polymorph, which saw 40% less nitrogen uptake than the *tbo* polymorph, due to a decrease in accessible surface area. The structural transformation of Ag-based one-dimensional coordination polymers was studied by Wright et al., whereby a different polymorph was observed following the loss of arene guest species [44]. Interestingly, the removal of these guest species resulted in a pair of polymorphs—one polymorph in the same one-dimensional architecture as the original coordination polymer, and one constructed in two-dimensions. Work carried out by Ward, Brammer and co-workers has illustrated the selective polymorph control of an Ag-based framework depending on alcohol adsorption [45]. Four polymorphs were observed in total, synthesised at high and low temperatures.

## Particle and macroscale structural control of MOFs

**Fig. 3:**
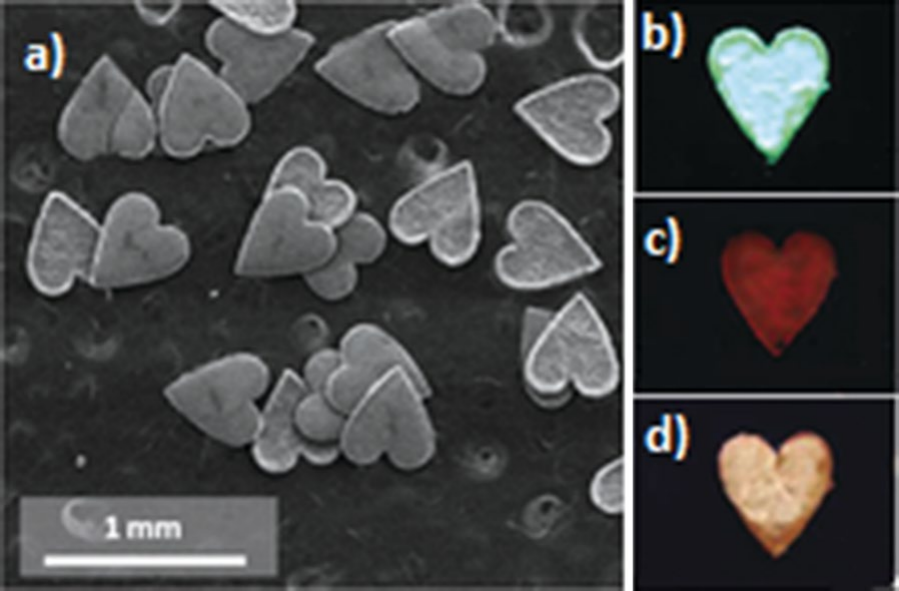
**a** SEM image of shape-controlled HKUST-1; **b**–**d** photographs of luminescent lanthanide MOF (LnBTC) (BTC = 1,3,5-benzenetricarboxylic acid) superstructures under exposure of UV light with wavelength of 265 nm; **b** green TbBTC, **c** red EuBTC, **d** apricot heterolanthanide MOF (Eu:Ce:Tb = 25:20:55) (Reproduced with permission from Ref. [48]. Copyright 2016 Wiley–VCH)

Interest in framework materials which lie outside the boundaries of traditional crystalline materials obtained from solvothermal methods has surged, and, similarly to the synthesis of MOFs in the gel-state described earlier in this review [19–21], examples of framework melting into a glass state are increasingly being reported. Bennett et al. explored the effect of melting a framework on the extended framework structure of a series of zeolitic imidazolate frameworks [46]. The connectivity was found to be maintained, though in a long-range disordered array. Following the transition of MOFs from a crystalline state to glasses, Thornton et al. probed the porosity change of melt-quenched MOF glasses [47]. Pore sizes of two related frameworks, ZIF-4 ([Zn(C_3_H_3_N_2_)_2_]) and ZIF-zni (formed from recrystallization of ZIF-4 to a known dense framework of identical chemical composition, obtained prior to melting), and the melt quenched glass *a*_g_ZIF-4, were determined experimentally and compared to simulated data. Interestingly, the sizes of the two pores of ZIF-4, 3.3 and 5.1 Å, did not alter proportionally, but instead to 2.6 and 6.9 Å respectively in ZIF-zni. Of particular note in the area of macroscale structural control is the synthesis of frameworks in controlled shapes and sizes. Kim et al. have developed a synthetic method based on interfacial interactions between an aqueous solution of metal salt and an organic linker solution [48]. Due to the immiscible nature of the solvent mixture, a micro-confiner mould is suspended on the interfacial surface. Within the spaces on the mould, shape controlled frameworks are synthesised, as seen in Fig. 3.

In conceptually related work, Carné-Sánchez et al. employed a spray-drying method to produce sub-5 µm hollow, spherical nanoscale MOFs, part of the class of frameworks known as nanoMOFs. Due to the innovative method of synthesis, the size and composition of these hollow MOF superstructures could be controlled. In total, 14 different spherical nanoMOFs, which include well-studied frameworks HKUST-1, MOF-74 and UiO-66, were synthesised using this novel approach [49]. This technique has since been adopted in the synthesis of other materials, including graphene oxide photocatalysts [50].

## Functionalisation

**Fig. 4:**
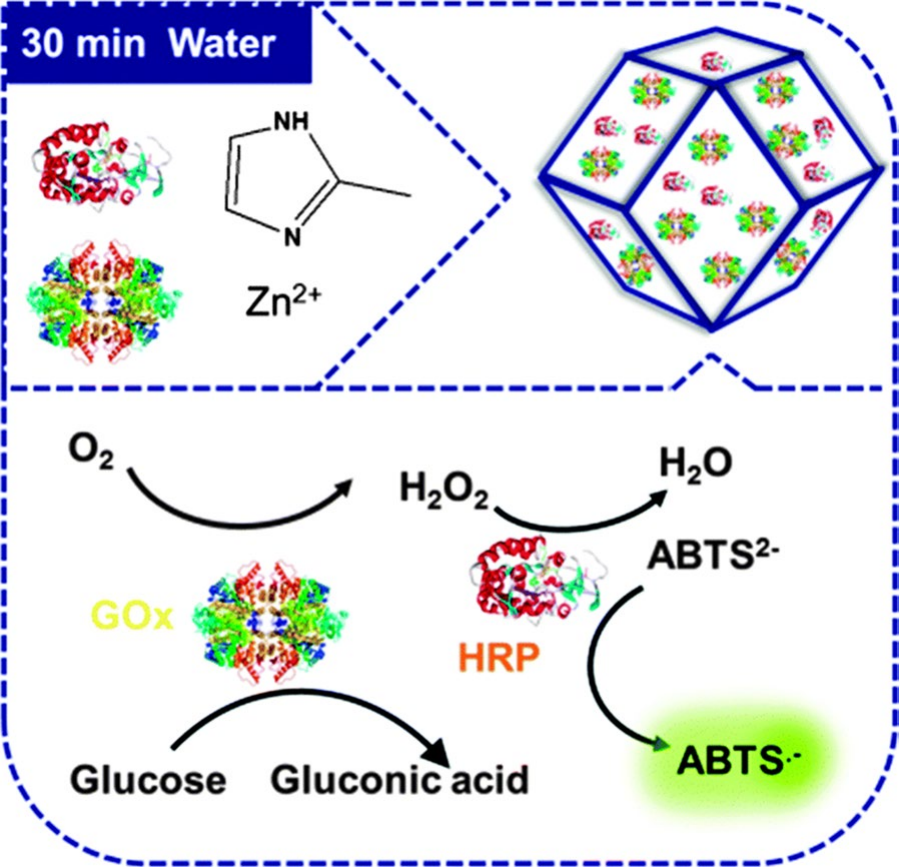
Schematic synthesis (top) and enzymatic cycle of multi-enzyme containing ZIF-8 (bottom) (Adapted from Ref. [51] with permission of The Royal Society of Chemistry)

In order to maximise the potential of metal–organic frameworks, it is commonplace to consider functionalisation of the framework itself. The tuneable structure of frameworks has, for many years, made them interesting candidates for catalytic applications, and, establishing a catalytically active system which is compatible with a biological environment, Ge and co-workers successfully constructed ZIF-8/glucose oxidase and horseradish peroxidase composite. The system, which can be seen in Fig. 4, catalyses the conversion of glucose to gluconic acid and ABTS^2−^ (2,2′-azino-bis(3-ethylbenzothiazoline-6-sulphonate) to ABTS^•−^ in aqueous solution at 25 °C [51]. The radical ABTS^•−^ is detectable at 415 nm, allowing for quantification of catalytic activity.

**Fig. 5:**
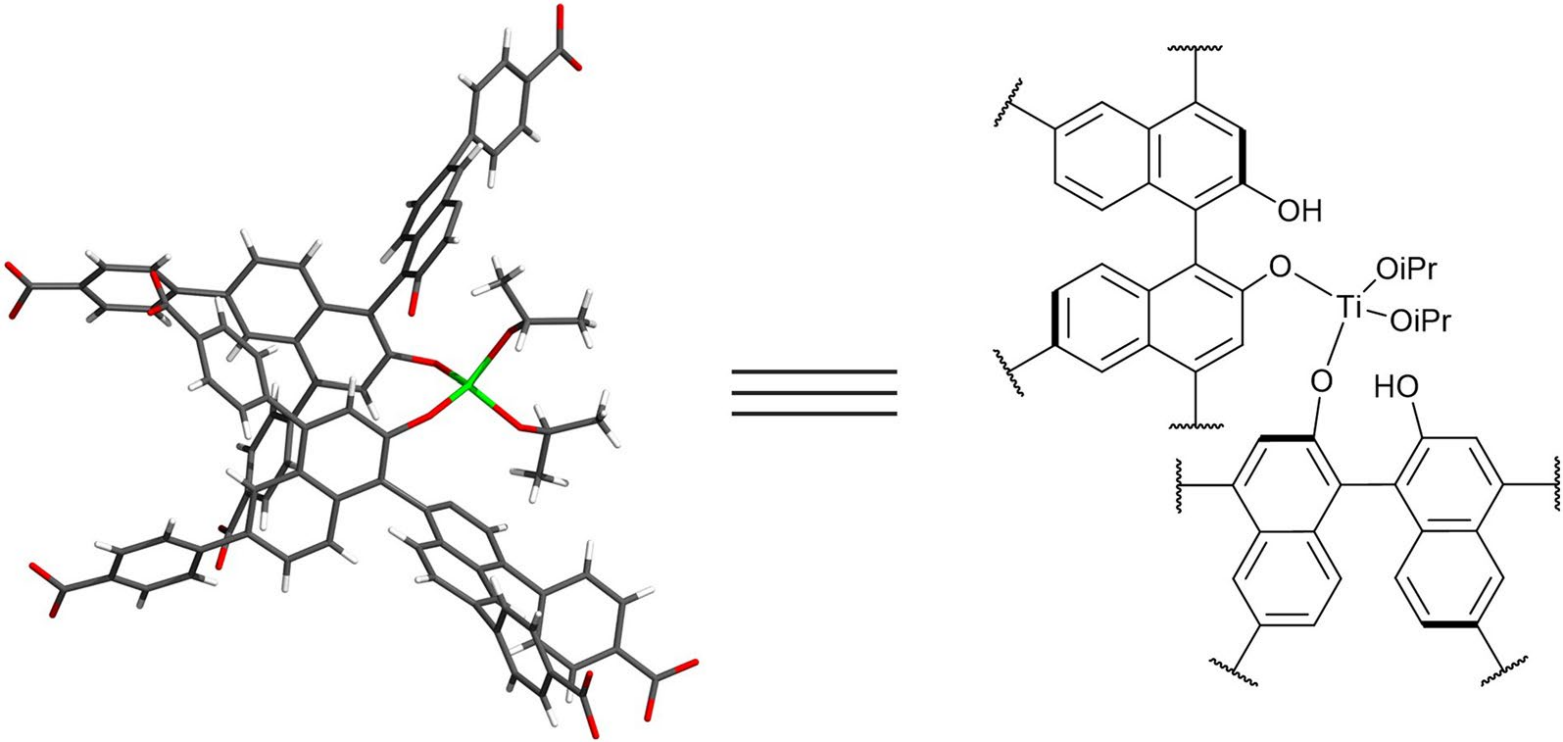
Single crystal x-ray structure (left) and ChemDraw structure (right) of the intermolecular [(OiPr)_2_Ti(BINOLate)_2_] species, where BINOL = [1,1′-binaphthalene]-2,2′-diol, formed following the introduction of Ti(OiPr)_4_. This is the species responsible for causing the interpenetration of the two networks (Adapted with permission from Ref. [52]. Copyright 2010 Wiley–VCH)

In 2010, Ma et al. investigated the effect of functionalisation of two interpenetrating chiral Zn-based MOFs on asymmetric catalysis [52]. Treatment of the frameworks with Ti(O*i*Pr)_4_ prompted the conversion of dihydroxy groups to Lewis acidic catalysts, as shown in Fig. 5, in one of the first examples of a framework being post-synthetically modified to form a catalytically active MOF. In one of the frameworks, this led to the crosslinking of two interpenetrating networks in a single-crystal to single-crystal conversion. Moderate enantioselectivity was observed for the addition of diethylzinc to aromatic aldehydes to afford secondary alcohols in the presence of the Ti-doped framework.

Similarly, work carried out by Wang et al. endeavoured to find a method for efficient post-synthetic modification (PSM) of a [Zn_2_(N_3_-BDC)_2_(dabco)], where dabco = 1,4-diazabicyclo[2.2.2]octane, surface-anchored thin film MOF [53]. They concluded that, commonly, PSM of framework surfaces is carried out via Cu-catalysed 1,3-dipolar cycloaddition, however removal of residual copper catalyst can be incredibly difficult, and cytotoxic Cu(I) ions minimise the potential applications that this method could have in life science or biological applications. Strain-promoted azide–alkyne cycloaddition (SPAAC), a metal-free click reaction, was successfully employed as an alternative PSM technique, to modify a pendant azide group on an aromatic linker with an eight-membered ring. The novel metal-free approach also saw near-quantitative modification of the surface, as monitored by infrared reflectance absorption spectroscopy (IRRAS) and powder X-ray diffraction (PXRD).

Post-synthetic modification is an incredibly useful technique to manipulate the function of frameworks, and work carried out by Aguilera-Sigalat et al. has developed a fluorescent pH sensor based on NH_2_-UiO-66. Constructed from octahedral Zr-nodes and 2-aminoterephthalic acid, the group post-synthetically modified the amino groups with an indole via a diazotisation reaction. The modification afforded increased stability of the framework in basic solutions, extending the accessible sensing range from pH 1 to pH 10 for unmodified NH_2_-UiO-66 to pH 1 to pH 12 for modified N≡N-UiO-66. The incorporation of light emitters into MOFs has been briefly reviewed by Furukawa and co-workers [54], who, in 2012, highlighted the synthesis of novel Eu-, Tb- and Eu/Tb-based frameworks by Cui et al. as use as luminescent thermometers [55]. These frameworks exhibit linear correlation between temperature and luminescence intensity from 50 to 200 K, with a 2,5-dimethoxy-1,4-benzenedicarboxylate linker acting as an antenna chromophore to sensitise Eu^3+^ and Tb^3+^ ions to effective energy transfer.

Another luminescent framework, [In_3_(btb)_2_(oa)_3_]_*n*_ (btb = 1,3,5-tris(4-carboxyphenyl)benzene, oa = oxalic acid) was described in 2012 by Nenoff and co-workers, who were seeking materials that can tune colour rendering index (CRI) and correlated colour temperature (CCT). Tunability of these colour properties is desirable for solid state lighting (SSL) [56]. The framework was found to emit white light, owing to broad-band emission over the entire visible light region. The study explored the effect that different concentrations of Eu^3+^-doping had on the colour properties of the framework, and observed an additional narrow red emission band following doping at three concentrations. Eu^3+^ was doped at 2.5, 5 and 10% relative to total indium content. Doping of the framework at the highest concentration afforded CRI and CCT values closest to those required for SSL applications.

Platero-Prats et al. have investigated functionalisation of a UiO-67 analogue with iridium complexes, and the effect that reaction time and relative acidity of the linkers present in the framework has on the extent of functionalisation [57]. The analogue is constructed from ZrCl_4_, BPDC and Ir–L (Ir–L = [Cp*Ir(bpydc)(Cl)Cl]^2−^, where Cp* = cyclopentadiene and bpydc = 2,2′-bipyridyl-5,5′-dicarboxylic acid), and by altering the amount of Ir–L metallated linker present in the reaction mixture, the structural dynamics of framework assembly could be probed. It was found that, after 12 h of reaction time, 50% of linkers present in the framework were metallated Ir–L, but this percentage decreased with longer reaction times. Interestingly, increased reaction times saw demetallation of the functionalised linker, and, subsequently, exchange of this linker with non-functionalised BPDC linker. Due to this, after 36 h of reaction time, the final framework contained less than half of the metallated Ir–L than frameworks yielded after 12 h of reaction time.

Another interesting example of framework functionalisation has been reported by Lu et al., where a chlorin-based framework, DBC-UiO (DBC = 1,5-di(*p*-benzoato)chlorin), proved to be an effective agent in photodynamic therapy (PDT) [58], which has shown great promise in cancer therapy. The framework was synthesised by reduction of the amino-functionalised terephthalic acid linker in previously prepared porphyrin-functionalised framework, DBP-UiO (DBP = 1,5-di(*p*-benzoato)porphyrin), to yield DBC-UiO. A red shift of the lowest-energy Q band was observed in the UV–vis absorption spectrum for DBC-UiO, which was 13 nm lower than in DBP-UiO, as well as DBC-UiO displaying an 11-fold increase in the extinction coefficient to 24,600 M^−1^ cm^−1^. DBC-UiO is also a photosensitizer with more efficient ^1^O_2_ generation than DBP-UiO, which accounts for its increased effectiveness in PDT.

Clearly, the incorporation of mixed materials, such as metal doping or ligand substitution into a framework, can affect the assembly process. There are examples, however, where this is not the case. Kang et al. showed that the incorporation of carboxyl-modified multi-walled carbon nanotubes (MWCNTs) into a JUC-32 framework did not alter the final framework structure or topology [59]. The resulting composite material was able to absorb more CO_2_ and CH_4_ per unit surface area than either material on its own. An example of mixed-metal framework synthesis in which the underlying framework structure is unchanged was reported by Schröder et al. in 2016, in which varied amounts of iron were doped into the synthesis of a gallium framework, MFM-300(Ga_2_) [60]. Doping of the framework with varying amounts of Fe^3+^ ions led to change in the gas adsorption capacities of the framework, with MFM-300(Ga_1.87_Fe_0.13_) showing the greatest change, affording a 49% increase in CO_2_ adsorption into the framework. Interestingly, synthesis of materials with higher levels of Fe^3+^ doping than described here led to the formation of irreproducible amorphous materials. Work carried out by Mali et al. in 2015 examined the distribution of linkers in a mixed biphenyl and bipyridyl dicarboxylic acid linker framework, through ^1^H and ^13^C solid state NMR (SSNMR) experiments [61]. This work was preluded by Kong et al. in 2013, who probed the distribution of functional groups in a mixed-linker framework constructed from six different linkers, using a combination of ^1^H, ^13^C and ^15^N SSNMR experiments, Monte Carlo and molecular dynamics simulations [62].

## MOF-templated phenomena

Due to the uniformity and tightly defined internal chemical environments of the pore structures, metal–organic frameworks have been used to template a growing variety of reactions. In 2012, Lin and co-workers demonstrated how a MOF-template strategy could be used to synthesise mixed metal oxide composites for use in photocatalytic reactions [63]. This straightforward method uses MIL-101(Fe) coated with amorphous titania to produce a material that can photocatalytically produce H_2_ from water; the individual components of the nanocomposite are unable to carry out this process alone. More recently, in 2015, MOF-545 was used to template the synthesis of 1D ultrafine metallic (Au and Pt) nanowires inside 1D pores controlling the morphology and dimensions of the metallic nanostructures that formed [64]. Also in 2015, Wang et al. described a method to synthesise metal hydroxides using a metal–organic framework template [65]. The Co-BPDC-MOF template was converted in an alkaline solution, replacing the carboxyl ligands with OH^−^ ions to give the porous cobalt hydroxide product. The cobalt MOF was chosen as a template due to the ease of its synthesis, and the transformation process that occurs via a solid–solid conversion, yielding a porous product with open diffusion channels. The templated Co(OH)_2_ demonstrated a superior performance with a specific capacitance of 604.5 F g^−1^ at 0.1 A g^−1^ and excellent rate capability and cycle stability. In another example, by Sun et al., magnetic nanoporous carbon (NPC) materials were synthesised using ZIF-67 as a template and carbon precursor [66]. ZIF-67 has a Co-based zeolitic imidazolate structure and is easily synthesised under ambient conditions; the magnetic MOF-derived materials are synthesised through thermal treatment of ZIF-67 at 1073 K, under a nitrogen atmosphere, yielding Co-ZIF-67. While NPCs are noted for their adsorbent properties, they can be difficult to separate from solution without centrifugation due to their small particle size. The introduction of magnetic hetero-metal particles in NPC materials increases ease of separation.

**Fig. 6:**
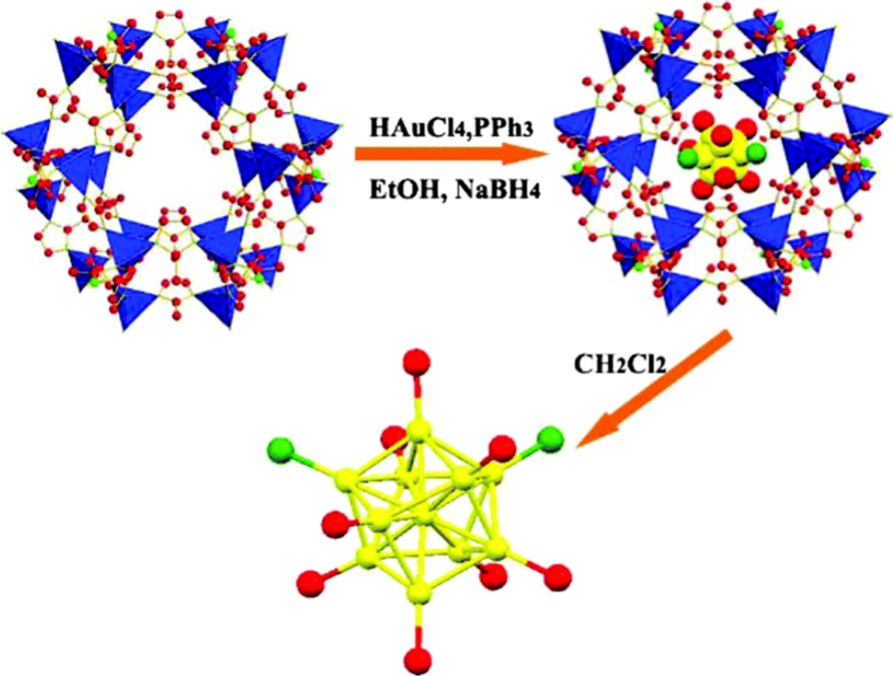
Schematic representation of Au NC synthesis in a framework and preparation of Au_11_NCs@ZIF-8 (Reproduced from Ref. [67] with permission from the Royal Society of Chemistry)

Recently, Lui et al. have reported the synthesis of atom-precise gold nanoclusters (NCs) by an in situ chemical reduction method of AuCl(PPh_3_) by sodium borohydride in ethanol, seen in Fig. 6, using MOFs as size-selection templates: ZIF-8 (Zn(MeIM)_2_, where MeIM = 2-methylimidazole) and MIL-101(Cr) ([Cr_3_F(H_2_O)_2_O(BDC)_3_]) [67]. The NC@MOF products were formed with high purity and exhibited catalytic behaviour for benzyl alcohol oxidation. This approach is highly promising for the formation of other NCs in size-selective synthesis using different frameworks of varying pore sizes.

MOFs have also been used as a template in the formation of LiFePO_4_ nanoparticles embedded in continuous interconnected N-doped carbon networks (LFP/N-CNWs) [68]. Liu and co-workers describe how MIL-100(Fe) can be used as both a porous template and source of iron and carbon starting materials by a carbothermal reduction reaction; this leads to a material of high surface area displaying excellent discharge capabilities due to the ease of Li^+^ and electron transfer.

## Organic polymers in MOFs

**Fig. 7:**
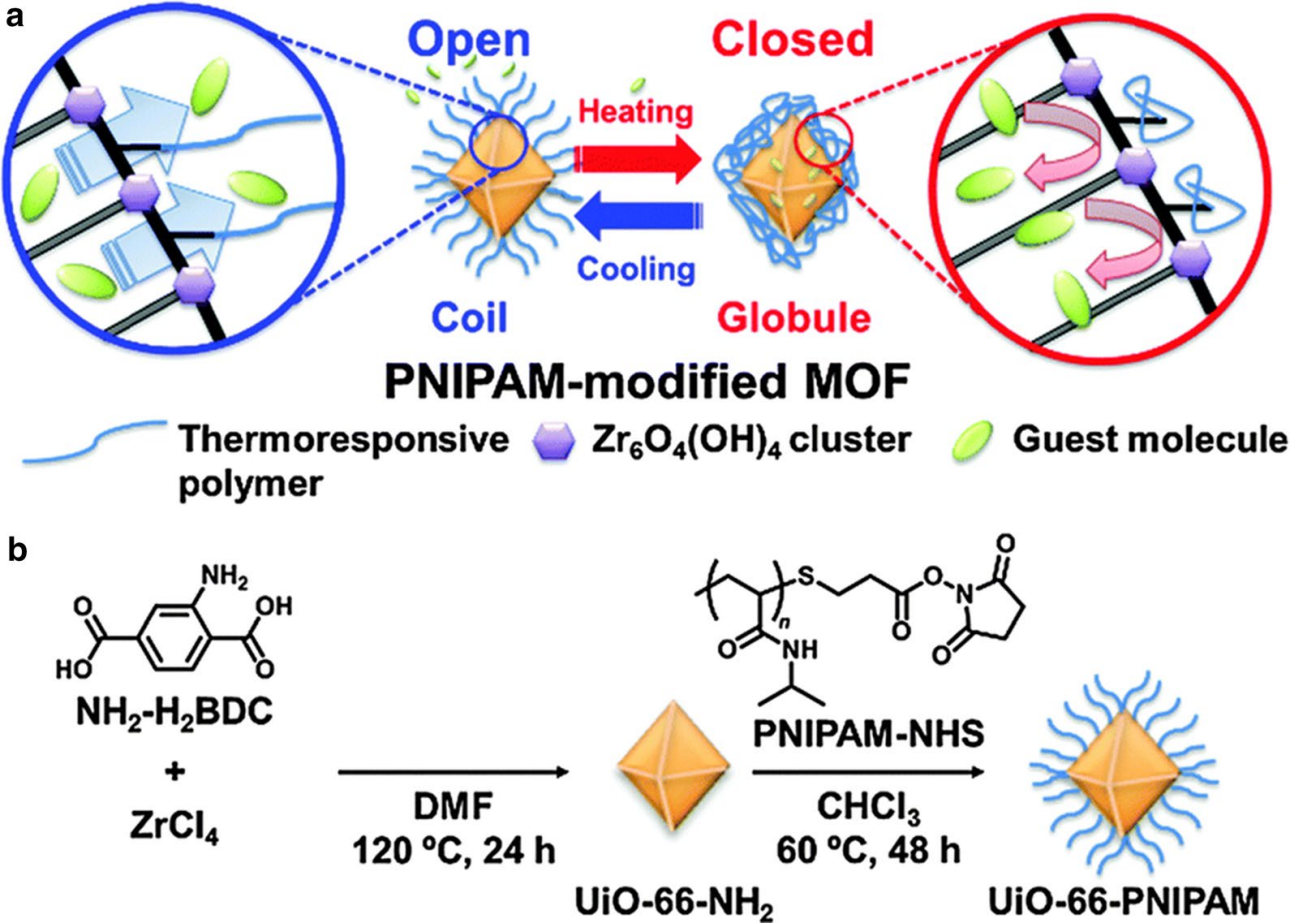
**a** Schematic image of controlled release using MOF tethering PNIPAM. **b** Preparation method of UiO-66-PNIPAM (Reproduced from Ref. [71] with permission from the Royal Society of Chemistry)

In 2005, Kitagawa and co-workers reported the first example of a radical polymerisation synthesis in the pores of a metal–organic framework [69]. The framework, [Zn_2_(BDC)_2_(triethylenediamine)]_n_, was soaked in a solution of monomer and initiator in order for them to penetrate into the pores, before being heated to induce polymerisation. This development has paved the way for further controlled polymerisations, and, by understanding the reaction mechanism of guest molecules, has allowed for the design of new frameworks for molecular confinement, alignment and conversion. A recent development in 2015 by McDonald et al. involved polymer grafting and coating on the surface of MOFs, leading to polymer hybridisation [70]. The approach used PSM of IRMOF-3 with a 2-aminoterephthalate linker to allow for the incorporation of tethered initiator sites. A “grafting from” method involved polymerisation from MOF active sites, allowing the polymer to grow from initiator sites. PSM of the MOF derived only from the 2-aminoterephthalate causes the initiator-carrying linker, and therefore the polymer, to be present throughout the framework, resulting in a substantial amount of pore space becoming blocked. In order to solve this problem, IRMOF-3 was grown onto the surface of MOF-5, which has a high surface area, forming IRMOF-3@MOF-5. Methyl methacrylate (MMA) was chosen as the monomer and underwent copper mediated atom transfer radical polymerisation to form PMMA@IRMOF-3@MOF-5. As the polymer chains are tethered to the outer shell of MOF-5, the high porosity is maintained. This particular method of grafting results in a complex polymer microstructure allowing for further development in the ability to modulate the accessibility of guests to a MOF. Post-synthetic modification of MOF surfaces has been achieved in a polymer-related approach, in which Nagata et al. modified the surface of a framework with a thermoresponsive polymer [71]. UiO-66 was modified with amino groups to give UiO-66-NH_2_, to which an amphiphilic polymer, PNIPAM (poly(*N*-isopropylacrylamide)), was covalently attached. The polymer is able to undergo a conformation change and can be switched through ON (open) and OFF (closed) states by lower and higher temperatures respectively, allowing for controlled release of guest molecules, which can be seen in Fig. 7.

## Host–guest chemistry in MOFs

The porous nature of metal–organic frameworks allows for a variety of host–guest chemistry. Yang et al. have neatly demonstrated the versatility of photoactive MOFs, carrying out the photopolymerization of a variety of photoactive guest molecules within the pores of a Mn-based framework, which also contains photoresponsive linkers [72]. When considering the photocatalytic properties of frameworks, Kataoka et al. synthesised a Ru(2,2′-bpy)_3_ (2,2′-bpy = 2,2′-bipyridine) framework which was capable of reducing water to hydrogen under visible light irradiation, in the presence of MV^2+^ (*N,N*′-dimethyl-4,4′-bipyridinium) and EDTA–2Na (where EDTA = ethylenediaminetetraacetic acid) [73]. Along related lines, Hupp, Farha and co-workers explored the photooxidation of a mustard-gas simulant using Zr-metalloporphyrin framework PCN-222 [74]. Singlet oxygen, ^1^O_2_, was generated by the photosensitized porphyrin linkers, which selectively oxidised the mustard-gas simulant to a non-toxic product. Similarly, work carried out by Mondloch et al. has probed the potential to use MOFs for the destruction of chemical warfare agents using Zr-based framework NU-1000 [75], where the framework acts as a catalyst for the hydrolysis of DMNP (dimethyl 4-nitrophenyl phosphate), a common nerve agent simulant. Yoon, Kim and co-workers have established that post-synthetic modification of amine-containing MOFs, to convert a tertiary amine to a quaternary *N*-alkyl ammonium salt, affords a framework that can separate differently charged organic dye molecules [76]. In another example of incorporating organic dyes in MOFs, Han et al. synthesised a new bimetallic framework, [(CH_3_)_2_NH_2_][Co_2_NaL_2_(CH_3_COO)_2_]·*x*S}_*n*_, (H_2_L = 5-(pyridine-4-yl)isophthalic acid) and investigated dye adsorption [77]. They found that smaller cationic dyes were readily adsorbed, while larger anionic and neutral dyes were hardly absorbed, indicating both a size- and charge-selective adsorption process.

**Fig. 8:**
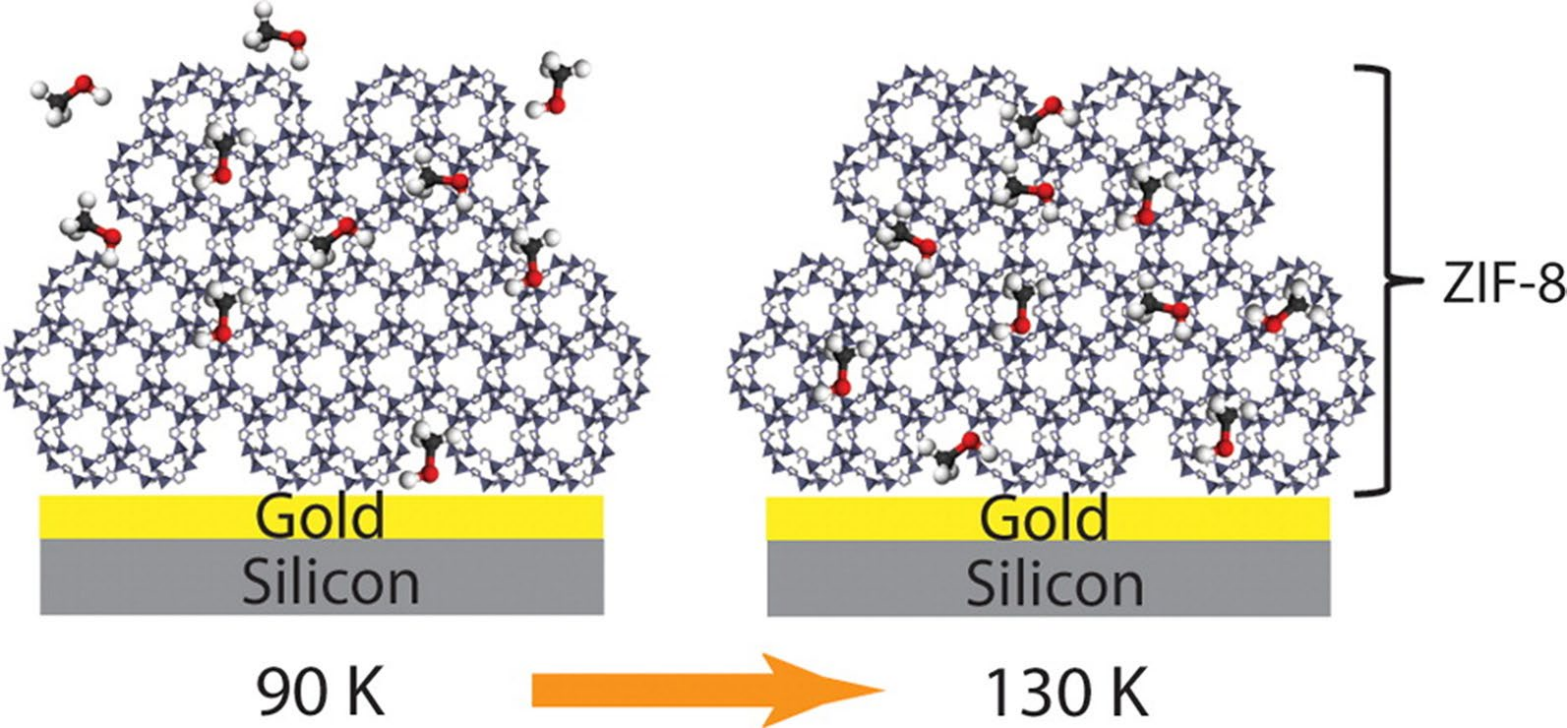
Ball-and-stick representation of methanol adsorption in ZIF-8 at different temperatures (Reprinted (adapted) with permission from Ref. [78]. Copyright 2016 American Chemical Society)

The adsorption of methanol into nanoparticle thin film ZIF-8 was explored by Mosier et al., where increased adsorption of the guest molecule was seen upon increase of temperature from 90 to 130 K, as shown in Fig. 8 [78]. Interestingly, this is contrary to the common behaviour of MOF materials, where guest adsorption generally decreases with increasing temperature. This work demonstrated the first example of controlled and monitored entry of guest molecules into a MOF film using temperature.

The acid gas stability of various frameworks was tested by Walton and co-workers, exploring the effects that exposure to each CO_2_, SO_2_ and water vapour had on the frameworks [79]. It was observed in transmission electron microscopy (TEM) images that exposure of MIL-125 to SO_2_ and H_2_O resulted in cavity defects along the edge of the crystallites, and similar exposure of CeBTC resulted in a softening of the particle edges. Contrastingly, an In-based framework reported by Savage et al. retains structural integrity following the binding and release of SO_2_, CO_2_ and N_2_, whilst the framework shows preferential binding towards SO_2_ [80]. In fact, the related Al-variant of the same framework, NOTT-300(Al) has very recently been shown to have long-term stability to SO_2_ exposure in a new “Long Duration Experiment” on I11, the powder X-ray diffraction beamline, at the Diamond Light Source [81].

When discussing stability of MOFs to different guests, water sensitivity of frameworks is not always an unwanted phenomenon; a Zn-based framework synthesised by Wang et al. was shown to be capable of the moisture-triggered controlled release of a common food flavouring and food preservative, allyl isothiocyanate [82]. Due to the presence of a Zn–N bond between the Zn-node and the nitrogen of the 4,4′-azobispyridyl linker, exposure of the material to moisture was able to hydrolyse the Zn–N bond, resulting in breakdown of the framework. Work carried out by Tamames-Tabar and co-workers has afforded a different Zn-framework, coined BioMIL-5 (Zn[C_9_O_4_H_14_]), exhibiting antibacterial effects [83]. These effects are due again to deliberate release of active constituents, azelaic acid and Zn^2+^ ions, following the breakdown of the framework. Bein and co-workers coated frameworks MIL-100(Fe) and MIL-101(Cr) with lipid bilayers, able to store dye molecules within the scaffold of the framework [84]. The lipid bilayer coating prevents the premature release of the dye molecules from the framework, which unlike the previous two examples, does not need to degrade to release the guest species. Due to the potential for pharmaceutical agent hosting shown by Bein and co-workers [84], Orellana-Tavra et al. have utilised amorphous UiO-66(Zr) as a host for the model drug molecule, calcein [85]. Comparisons were made between the amorphous and crystalline forms of UiO-66, and the amorphous material was found to sustain release of calcein for up to 30 days, compared with the 2 days afforded by the crystalline counterpart. In comparison, Lin et al. have loaded anti-cancer drug methotrexate into Zr-based porphyrin framework PCN-221 [86]. High drug loading and pH-responsive release was observed, allowing for limited drug release in undesirable biological areas. Following pH initiated release, quick loss of methotrexate was observed after 8 h, followed by slow dissolution. A currently less explored stimulus for MOF-guest release is photoresponse. Hill and co-workers have explored this, by coating optical fibres with UiO-66, and subsequently loading this framework with the anticancer drug 5-fluorouracil (5-FU) [87]. To counteract the commonly encountered issue in oncological therapies of drug release outside of the target area, photostimulated guest release was utilised. Irradiation of the framework, via the optical fibre, at 1050 nm, sufficiently activated UiO-66 in order to overcome the enthalpy of adsorption for 5-FU. No guest drug was detected in the test solution prior to irradiation.

## Sensing with MOFs

Metal–organic frameworks displaying sensing properties have been prevalent in recent years. An example of MOFs being incorporated into a working sensor was reported in 2011 by Han et al., in which they describe a method of wet stamping whereby micropatterns of several organic chemicals are imprinted into the crystals of MOF-5 and CD-MOF-2 (formed from γ-cyclodextrin and rubidium hydroxide) [88]. This technique means that the frameworks can react to external conditions (pH change, light exposure, etc.) and the imprinted chemicals can change colour or appearance as a response. Monitoring luminescence emission is a common method for sensing and detection. In 2014, a ratiometric fluorescent pH sensor was developed by Lu and Yan, using assembly of a lanthanide complex with β-diketonate, which is attached to MOF-253 through post-synthetic modification MOF-253. There are two types of Eu^3+^ in the framework, with different characteristic excitation wavelengths, and only one is sensitive to pH. Therefore, this pH sensor shows promise for applications in biomedical research, and as it requires no calibration in the pH range 5.0–7.2 it is suitable for studies in biological fluids [89].

Computational density functional theory (DFT) and time-dependent DFT studies have been used to investigate the sensing applications of MOFs, whereby Zhao et al. looked at the possible interactions of formaldehyde with a luminescent metal–organic framework, [Zn_2_(H_2_L)(2,2′-bpy)_2_(H_2_O)]_n_ where L = 3,3′,3′-[1,3,5-phenylenetri(oxy)]triphthalic acid, through the formation of hydrogen bonds [90]. Other examples of luminescent sensors have been experimentally investigated, such as five new lanthanide frameworks with flexible linkers by Wang et al. [91]. Of those synthesised, they found that [Eu_2_L_2_(H_2_O)_3_]·2H_2_O, where L = 1,3,5-tris(4-carboxy-phenyl-1-ylmethyl)-2,4,6-trimethylbenzene, was able to sense small organic molecules like acetone, and aromatic compounds like nitrobenzene. These compounds were found to significantly quench luminescent intensity, and in particular, those containing functional groups such as hydroxyl groups that can interact with fluorophores through electrostatic interactions, meant that the quenching effect could be maintained over a long range due to the energy transfer mechanism. The analogous Yb framework showed selective adsorption of carbon dioxide over nitrogen and methane making it of interest for potential gas separation applications.

A growing area of interest is the detection of molecules with military significance. Nitroaromatics are a well-known class of explosive compounds, as well as pollutants, that have also been detected by luminescent MOFs. A lanthanide-containing framework, [Tb(L_1_)_2/3_(BDC)_1/2_(H_2_O)_2_]·2H_2_O (where L_1_ = 2,4,6-tris(4-carboxyphenoxy)-1,3,5-triazine), has been reported which shows strong luminescence emission for detection of these compounds, which is easily observable under a UV lamp. This has many advantages over well reported d^10^ (Zn or Cd) transition metal frameworks which show weak, non-characteristic luminescence behaviour [92]. Green emission at 545 nm of Tb-MOF arises from the highly conjugated structure of the ligands acting as “antenna”, increasing the optical performance of the lanthanide centre. The luminescence was quenched by nitroaromatics, and interestingly, the photoluminescence was found to be regained upon washing the Tb-MOF sample with ethanol. Qin et al. also explored the detection of nitroaromatics with a different terbium framework, which upon activation shows a high selectivity for these molecules in aqueous and vapour phases [93]. Although not strictly a pure sensing application, the work of Hupp and Farha on chemical weapon decontamination is noteworthy and one example has already been referenced above [74]. Subsequent work in the group by Moon et al. has examined the detoxification of chemical weapon agents (CWAs) GD and VX [94], as well as the simulant dimethyl 4-nitrophenylphosphate (DMNP), using a Zr-based MOF/polymer mixture in aqueous solution.

Fluorescence sensing using MOFs also proves useful for detection of biological entities. Chen and co-workers designed a copper framework, [H_2_dtoaCu] where H_2_dtoa = *N,N*-bis(2-hydroxy-ethyl)dithiooxamide, that can be used for the sequence-specific recognition of duplex DNA [95]. A triplex-forming oligonucleotide labelled with fluorescein amidite (FAM) was used as a probe; fluorescence quenching (Q_E_ = 88.7%) was observed as a result of a photoinduced electron transfer process due to chemisorption of the FAM dye by the framework. This effect was reversible and fluorescence could be recovered, due to the target ds-DNA releasing the probe.

DNA can also be detected electrochemically, as described by Ling et al. in 2015, whereby an extremely sensitive sensor was developed by incorporating the electrocatalysis of a streptavidin (SA) functionalised Zr-porphyrin MOF, PCN-222@SA, with a triple-helix molecular switch for signal transduction. Exonuclease III was also used for signal amplification to improve the sensitivity giving a DNA detection limit of 0.29 fM [96]. Protein detection has been investigated using MOFs, in which they are combined with molecular imprinting and upconversion nanoparticles (UCNPs) [97]. Guo et al. chose to use HKUST-1, [Cu_3_(BTC)_2_], with a very high specific surface area, to create a fluorescent and stable composite material with the UCNPs. An imprinting method was used to create a thermo-sensitive layer consisting of bovine haemoglobin as a template and *N*-isopropyl acrylamide as a functional monomer which can change in size as a response to temperature. The rate of mass transfer and adsorption capacity was increased upon incorporation of MOFs when compared to common molecularly imprinted polymers (MIPs). The fluorescence intensity of the composite UCNP/MOF/MIP was seen to decrease with an increasing haemoglobin concentration, and successful thermo-sensitivity was observed for specific recognition of proteins.

**Fig. 9:**
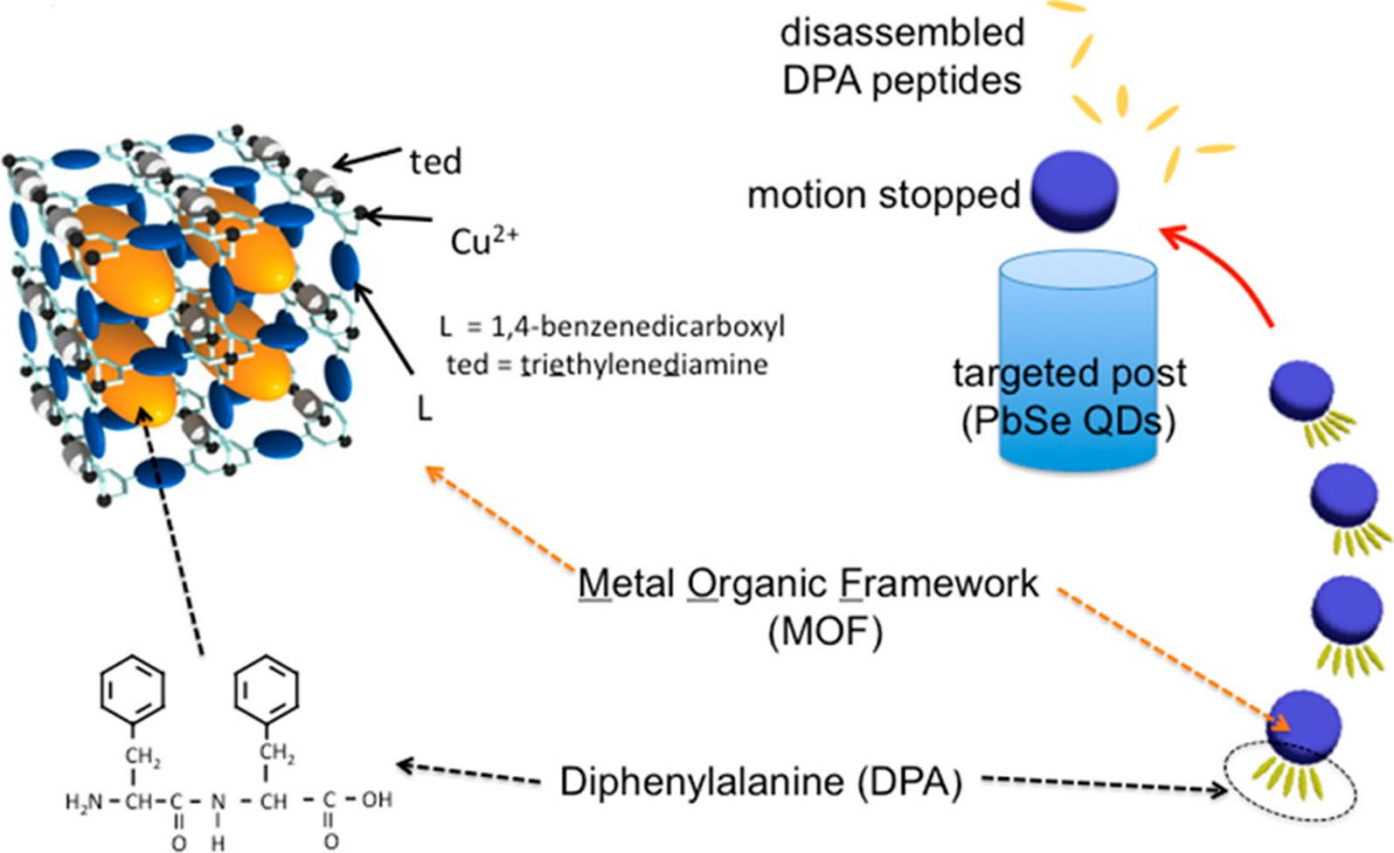
Scheme depicting peptide-MOF motor swimming toward high pH. The reassembly of released hydrophobic DPA peptides on the edges of the framework produces an asymmetric surface tension distribution that powers motion towards the higher surface tension side (left). A change of pH gradient in environment prompts completion of the motion due to higher pH conditions disassembling DPA peptides on the MOF (right) (Reprinted (adapted) with permission from Ref. [98]. Copyright 2015 American Chemical Society)

Instead of using colour or fluorescence variation, Ikezoe et al. exploited a rather different method of reporting on environmental changes by developing synthetic ‘swimmers’ that are sensitive to chemical gradients on the macroscale (Fig. 9) [98]. CuJAST-1 ([Cu_2_BDC_2_ted]_n_, where ted = triethylenediamine) was chosen because of its excellent storage ability of the peptide fuel, and by integrating it into the swimmers motor component allowed for the detection of heavy metal ions. Specifically, a lead-binding urease enzyme was chosen to conjugate to PbSe quantum dots encouraging the peptide-MOF to swim towards such targets. The diphenylalanine (DPA) peptide can be released from the pores of the MOF and undergoes a robust self-assembling process at the edge of the MOF which induces an asymmetric surface tension distribution, triggering motion towards the higher surface tension side of the particle. The motion of MOF motor slows as it moves closer to highest gradient point, and eventually stopping at the region of highest Pb-concentration. Particles of this design are generally limited to movement in one direction. Nevertheless, this is an unusual and highly visual method of interrogating otherwise invisible concentration gradients in dynamic solutions and is an imitative example of chemotaxis that can direct the motion by sensing the location of target; this indicates potential for further interesting developments in the future.

## Electroactive MOFs

With the electronic properties of metal–organic frameworks having received little attention, D’Alessandro and co-workers published one of the first examples of a redox active material in 2012 [99]. The redox properties of [Zn_2_(NDC)_2_(DPNI)], where NDC = 2,7-naphthalene dicarboxylate, DPNI = *N,N*′-di(4-pyridyl)-1,4,5,8-naphthalenetetracarboxydiimide, were studied using solid state cyclic voltammetry (CV), whilst the optical properties of the framework were investigated using an in situ UV–Vis-NIR spectroelectrochemical (SEC) technique. A different zinc redox-active framework was published the following year by Leong et al. in which electron paramagnetic resonance (EPR) measurements were able to show the photogeneration of the paramagnetic radical states of the material [100]. More recently, D’Alessandro reported three cobalt frameworks, in which the degree of interpenetration was controlled whilst retaining the redox-active properties of the tris(4-(pyridin-4-yl)phenyl)amine linker [101].

MOFs have begun to show potential in more uncommon applications such as electrochemical devices. Redox-active organic linkers that can change colour as a response to an electrochemical stimulus are a crucial part of electrochromic frameworks, such as one of the first reported examples by Wade et al. in 2013. They developed Zn-pyrazolate frameworks with core-substituted naphthalene diimide (NDI) linkers, similar to the work of that described by D’Alessandro. The frameworks, [Zn(NDI-X)] where X = H, S-C_2_H_5_ or NH-C_2_H_5_, were deposited on fluorine-doped tin oxide (FTO) surfaces [102]. The films displayed electroactive behaviour with rapid, reversible colour switching, which was found to coincide with reduction events during electrochemical cycling. Also reported in 2013, was another electrochemically active MOF film, consisting of acicular (needle-shaped) nanorods, in which there is a reversible colour switch between yellow and deep blue as a result of a one-electron redox process at the pyrene units situated on the pyridine-based linkers [103]. Another electrochemical use of MOFs is that of energy storage, as displayed by Shrestha, Han and co-workers, in which a cobalt framework film was deposited on an ITO (indium tin oxide) substrate. They found that the material exhibited pseudocapacitor behaviour with reversible electrochemical switching, leading to possibilities for further exploration of MOFs being used in electrochemical devices [104]. Finally, in another example of combining key features of electrochemistry and metal–organic frameworks, Hod et al. reported the electrophoretic formation and growth of four well-known MOFs: NU-1000, UiO-66, HKUST-1 and MIL-53(Al) [105]. As the MOFs studied contain defects, there is some partial charge on the surfaces. The method of electrophoretic deposition (EPD) drives the charges to the oppositely charged electrode and was found to drive MOF deposition, allowing for the assembly of micropatterned films. The results indicated the importance of properties such as charge transport and electrical conductivity, allowing for synthesis of complex, multi-functional surface constructions with multiple MOF films by EPD.

## Conclusions

Increasingly, the niche areas of MOF science are being recognised as having enormous potential [106]. Looking beyond the gas uptake capabilities of metal–organic frameworks that have been dominating the literature so far this century, this review has detailed a relative handful of the varied and alternative applications for these tuneable porous materials. Many of the examples used throughout this review demonstrate that the extant boundaries between material applications are becoming increasingly blurred. A prime example of this boundary-crossing is that of a thin-film SURMOF (surface-mounted metal–organic framework) used to template polymer formation with applications for drug loading, published just this year [107]. It is also evident that applications for MOFs in chemical sensing are becoming increasingly important, with this important growing niche having been ably reviewed this year by Ghosh and co-workers [108]. The interaction of guest molecules with frameworks has led to a large number of the unusual properties discussed herein: from the treatment of chemical weapon analogues [109] to phototriggered release of carbon monoxide [110], to the effects of guest loading on structure templating and molecular separations [111]. Metal–organic frameworks are famous for their tunability, and while the ability to ‘design’ a framework structure or function is far better developed that in previous years, it can still be difficult to predict the behaviour that guest molecules will display within a framework. Understanding such dynamic host–guest behaviours is critical when considering framework design if a specific application is sought, and represents one of the greatest challenges facing the field at this time.

## Footnotes

1 Based on a Web of Science search performed on 15/05/2017 using the search term “metal organic framework” (including quotation marks), refined to articles published since 2012.
